# QuickStats: Percentage* of Children and Adolescents Aged 5–17 Years Who Received Free or Reduced-Cost Meals at School During the Previous 12 Months,^†^ by Race and Hispanic Ethnicity^§^ and Family Income^¶^ — National Health Interview Survey, United States, 2021

**DOI:** 10.15585/mmwr.mm7220a6

**Published:** 2023-05-19

**Figure:**
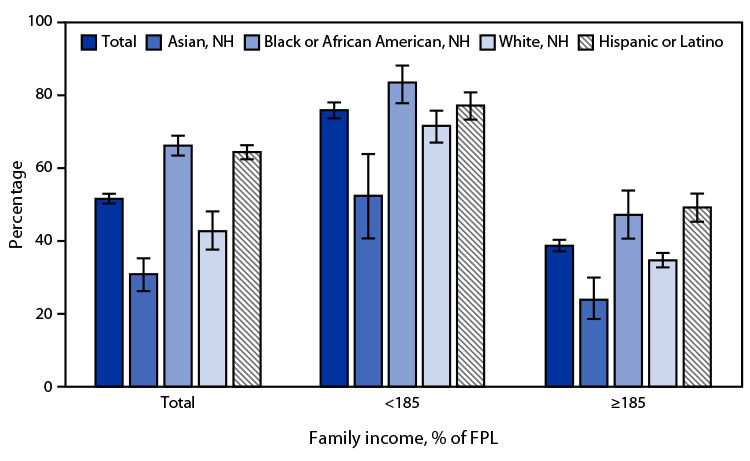
In 2021, 51.6% of all U.S. children and adolescents aged 5–17 years received free or reduced-cost meals at school during the previous 12 months; NH Black or African American (66.2%) and Hispanic or Latino (Hispanic) (64.4%) children and adolescents were more likely to receive free or reduced-cost meals at school than were NH White (42.7%) children and adolescents, with NH Asian (30.9%) children and adolescents having the lowest percentage. The same pattern was observed for children and adolescents in families with income ≥185% of the FPL, but the observed difference in receiving free or reduced-cost meals between Hispanic and NH White children and adolescents was not significant for the lower-income group. Children and adolescents in families with incomes <185% of the FPL were more likely to receive free or reduced-cost meals compared with children and adolescents in families with incomes ≥185% of the FPL (75.9% versus 38.7%).

**Source:** National Center for Health Statistics, National Health Interview Survey, 2021. https://www.cdc.gov/nchs/nhis.htm

